# Comprehensive molecular characterization of craniopharyngiomas using whole transcriptome and spatial transcriptomics approaches

**DOI:** 10.1007/s10014-025-00509-z

**Published:** 2025-07-09

**Authors:** Špela Kert, Alenka Matjašič, Jože Pižem, Jernej Mlakar, Matic Bošnjak, Miha Jerala, Primož Kotnik, Barbara Faganel Kotnik, Lidija Kitanovski, Andrej Zupan

**Affiliations:** 1 Institute of Pathology, Faculty of Medicine, University of Ljubljana, Ljubljana, Slovenia; 2 Clinical Institute of Genomic Medicine, University Medical Centre Ljubljana, Ljubljana, Slovenia; 3 Faculty of Medicine, University of Ljubljana, Ljubljana, Slovenia; 4 Department of Endocrinology, Diabetes, and Metabolic Diseases, University Children’s Hospital, University Medical Centre Ljubljana, Ljubljana, Slovenia; 5 Department of Hematology and Oncology, University Children’s Hospital, University Medical Centre Ljubljana, Ljubljana, Slovenia

**Keywords:** Craniopharyngioma, Transcriptional analysis, Somatic mutation detection, Differential gene expression, In situ spatial profiling

## Abstract

Craniopharyngiomas (CPs) are rare benign brain tumors that are classified as WHO grade I, with two subtypes: adamantinomatous craniopharyngioma (ACP) and papillary craniopharyngioma (PCP). ACP is caused by somatic mutations in exon 3 of the *CTNNB1* gene activating the Wnt signaling pathway. PCP is associated with somatic *BRAF* p.V600E mutations activating the MAPK signaling pathway. Understanding their molecular differences is crucial for diagnosis and treatment. This study aimed to analyze common somatic alterations in ACP and PCP using bulk transcriptome sequencing and in situ spatial transcriptomics. RNA sequencing and high-resolution spatial profiling were used to detect mutations and examine gene expression differences among ACP, PCP, and healthy pituitary tissue. Whole transcriptome sequencing was performed on 24 tumor samples, with healthy pituitary data from the GTEx portal. Bioinformatics analysis utilized the CTAT mutation pipeline, with Sanger sequencing for validation. Results confirmed *BRAF* p.V600E mutations in all PCP samples and *CTNNB1* mutations in all ACP samples. Differential gene expression analysis highlighted distinct molecular profiles and reinforced the involvement of Wnt and MAPK signaling. Spatial profiling identified 41 differentially expressed genes between ACP and PCP. This study provides critical insights into CP biology, supporting improved diagnostics and potential therapeutic strategies.

**Supplementary Information:**

The online version contains supplementary material available at 10.1007/s10014-025-00509-z.

## Introduction

CPs are rare benign brain tumors classified by the World Health Organization (WHO) as central nervous system (CNS) WHO grade I [43]. Due to their location, a growing tumor can press on the pituitary gland and hypothalamus and impair their function. There are two recognized histologic types: adamantinomatous craniopharyngioma (ACP) and papillary craniopharyngioma (PCP) [28, 29, 33]. ACP exhibits a bimodal age distribution and occurs in children and later in adults aged 40–60 years, whereas PCP occurs mainly in adults [28, 29, 33]. Despite being benign, they are associated with a poor quality of life due to their impact on surrounding tissues, and require aggressive therapy, including surgery or radiotherapy [17, 22, 29].

ACP is characterized by mixed solid and cystic epithelial tumors with distinct histologic features, including stellate reticulum, calcifications and wet keratin [42, 43]. Somatic mutations in exon 3 of the *CTNNB1* gene are driver mutations responsible for the development of ACP [17, 32, 40, 42, 43]. In some cases, germline mutations in the *APC* gene are thought to be responsible for tumor development [19, 43]. These mutations lead to the activation of the Wnt signaling pathway, resulting in excessive cell proliferation, invasion and tumor development [17, 32, 40, 42, 43]. PCP is associated with somatic mutations in the *BRAF* gene, with almost all cases harboring the *BRAF* p.V600E mutation [28, 32, 40, 43]. This mutation activates the MAPK (mitogen-activated protein kinase) signaling pathway, which is a known mechanism of cancerogenesis [28, 32, 40, 43].

Traditionally, somatic mutations have been detected by DNA sequencing of tumor tissue [24, 34]. Identification of these mutations is essential for characterization of the cancer genome and accurate classification of the tumor [34]. However, transcriptional analysis provides an alternative method for detecting somatic mutations, including rare exonic somatic mutations [34, 44]. This approach not only identifies somatic mutations but also enables the detection of fusion transcripts, exon splicing events, adapter clipping and RNA editing [34]. Importantly, formalin-fixed paraffin-embedded (FFPE) samples, which are commonly used in routine clinical settings, can be used for transcriptional analysis [8, 30, 37, 38]. Although processing FFPE samples can degrade and chemically modify RNA, advances in extraction and sequencing technologies have improved the recovery of usable RNA, allowing meaningful gene expression studies from these samples [8, 30, 37, 38].

Xenium is a spatial transcriptomics technology that efficiently maps the spatial distribution of transcripts within tissue sections. This is an effective solution to the challenge of spatial information loss associated with bulk RNA sequencing. By providing high-resolution, in situ spatial profiling, Xenium allows us to observe the localization of gene expression within the tumor microenvironment. This capability is particularly valuable for understanding the intricate cellular interactions and heterogeneity within tumors, which is essential for developing targeted therapeutic strategies. The combination of traditional RNA sequencing with innovative technologies such as Xenium enables a more comprehensive characterization of tumors at both the genetic and spatial levels.

In this study, bulk and spatial transcriptome methods, including Xenium spatial transcriptomics, were used to examine CPs cases. The objectives were to identify common somatic alterations, evaluate the utility of transcriptomic data in routine diagnostics, and discover novel biomarkers. In addition, transcriptional analysis compared gene expression among ACP, PCP and normal pituitary tissue, providing deeper insights into the molecular pathogenesis of CPs.

## Materials and methods

### Tumor samples

A total of 24 tumor samples, collected between 2012 and 2022, were included in this study. Diagnoses were established by a neuropathologist based on hematoxylin and eosin (HE) staining, with some cases further confirmed by immunohistochemical (IHC) staining for β-catenin (for ACP) or a BRAF p.V600E mutation (for PCP).

In the PCP group, the mean age was 48.0 years (SD ± 11.3). A binomial age distribution was observed in the ACP group. For pediatric ACP cases, the mean age was 7.3 years (SD ± 3.7), while in the adult ACP cases, the mean age was 48.4 years (SD ± 19.6). Tumor samples were formalin-fixed and paraffin-embedded (FFPE) and stored at room temperature until RNA isolation. One ACP sample (ACP_12) was excluded from the analysis due to excessive calcification, which compromised the tissue quality.

### HE and Immunohistochemistry

We performed IHC staining for β-catenin and BRAF p.V600E in an automatic immunostainer Benchmark XT (Ventana Medical Systems Inc., Tucson, AZ). We used anti-BRAF (VE1) antibody (cat no. ab228461, 1:200, Abcam plc, Cambridge, UK) and Beta-Catenin (14) antibody (cat. no. 224M-15, 1:20, Cell Marque, Rocklin, CA, USA). Sections were treated with biotinylated secondary antibody and incubated with peroxidase conjugated streptavidin (iVIEW DAB Detection Kit, Ventana Medical Systems Inc., Tucson, AZ), according to the manufacturer’s instructions.

### RNA extraction, library preparation and sequencing

We extracted RNA from archived FFPE tissue samples using a Promega Maxwell RSC RNA FFPE Kit (Promega Corporation, Madison, WI, USA) according to the manufacturer’s instructions. We prepared cDNA libraries using a QIAseq Stranded RNA Library Kit (QIAGEN, Maryland, USA) according to the manufacturer’s instructions. RNA sequencing was performed on the NovaSeq platform from Illumina (Illumina, San Diego, CA, USA). For each sample, an average of 50 million reads were collected in the form of FASTQ files. We trimmed the raw reads using the fastp tool [6]. We mapped the trimmed reads to the human transcriptome using the STAR aligner [10] and obtained gene counts with the quantMode option in STAR.

### Mutation detection and differential expression analysis

We identified somatic mutations using the CTAT mutation pipeline (https://github.com/TrinityCTAT/ctat-mutations). In three cases in which transcriptional analysis detected a driver mutation in the *CTNNB1* gene with a low frequency, we performed Sanger sequencing of exon 3 of the *CTNNB1* gene. We conducted Sanger sequencing at both DNA and RNA levels. For the RNA samples, we first performed reverse transcription using an IonTorrent NGS Reverse Transcription Kit (Thermo Fisher Scientific, Massachusetts, USA). The primers we used are listed in Supplementary Table 1.

For PCR amplification, we employed specific primer pairs designed with the PrimerQuest Tool (Integrated DNA Technologies, Coralville, IA) and FastGene Optima HotStart ReadyMix (Nippon Genetics Europe, Dueren, Germany), according to the manufacturers’ instructions. We visualized the PCR products on a 2% agarose gel to determine size and specificity. We then purified PCR products using an ExoSAP-IT enzymatic approach (Applied Biosystems, Foster City, CA).

For Sanger sequencing, we used 2 μL of the purified PCR product, 1 μL of the specific forward or reverse primer and a BigDye Terminator Cycle Sequencing Kit 3.1 (Thermo Fisher Scientific, Massachusetts, USA). We cleaned the Sanger sequencing products with a BigDye XTerminator purification kit (Thermo Fisher Scientific, Massachusetts, USA) according to the manufacturer's instructions. We performed Sanger sequencing with the SeqStudio (Thermo Fisher Scientific, Massachusetts, USA), and analyzed the sequences using the SeqScanner software (Thermo Fisher Scientific, Massachusetts, USA) and the Ensemble Genome Browser [16].

We performed differential gene expression analysis using RNASeqChef [12] with the standard DESeq2 protocol [27]. The analysis included 13 ACP samples, 4 PCP samples and 49 healthy pituitary samples. Raw reads were normalized using DESeq2, and rlog transformation was performed. We used Seaborn [41] to obtain barplots. Statistical differences were calculated using the Mann–Whitney *U* test.

### Targeted DNA sequencing library preparation

Prior to library preparation, we extracted DNA from archived FFPE tissue samples using a Promega Maxwell RSC DNA FFPE Kit (Promega Corporation, Madison, WI, USA) according to the manufacturer’s instructions. We treated the extracted DNA samples with uracil-DNA glycosylase (UDG) enzyme to remove uracil residues. We used 15 ng of UDG-treated DNA and an AmpliSeq Library kit Plus (Thermo Fisher Scientific, Massachusetts, USA). We performed thermal cycling according to the manufacturer’s recommendations, which included 18 cycles for target amplification. We performed all further steps (amplicon digestion, adapter ligation and library cleaning) according to manufacturer’s instructions. For the gene list associated with the OCCRA panel, refer to Oncomine™ Childhood Cancer Research Assay (thermofisher.com).

### Spatial transcriptomic analysis of PCP and two ACP samples

We prepared three tissue samples for Xenium analysis. We used the 10 × Genomic panel “Human Multi-tissue and Cancer,” (PN-1000626, 10 × Genomics), with which we analyzed expression levels of 377 genes.

The Xenium workflow began with the preparation of FFPE tissue for specific Xenium slides, according to the “Xenium In Situ for FFPE-Tissue Preparation Guide” (CG000578 Rev C, 10X Genomics). The tissue was processed following the “Xenium In Situ for FFPE-Deparaffinization and Decrosslinking” protocol (CG000580 Rev C, 10X Genomics) and selected probes were hybridized to the RNA targets according to the “Xenium In Situ Gene Expression – Probe Hybridization, Ligation & Amplification User Guide” protocol (CG000549 Rev A, 10 × Genomics). On the Xenium platform, the tissue was sequenced with amplified probe products and onboard image data processing was performed to identify and localize the individual transcript targets. Finally, post-run H&E staining was performed following the “Xenium In Situ Gene Expression – Post-Xenium Analyzer H&E Staining” protocol (CG000613 Rev B, 10 × Genomics).

We started the downstream analysis with an overview in Xenium Explorer 2 software [21]. We obtained the number of transcripts for each of the 377 genes for each section individually. The transcripts were then normalized based on the number of cells as the number of transcripts per 1000 cells for each individual section per gene. We then performed standard DESeq2 [27] analysis to identify differentially expressed genes between PCP and ACP. We also performed an automatic cluster annotation with community-available Seurat toolkit [14], which also provides support for Robust Cell Type Decomposition, a computational approach for deconvoluting spot-level data from spatial datasets when annotated with a scRNA-seq reference. We used Allen Brain Map [11] RNA-Seq Data: Human MTG 10 × SEA-AD [1], whereby we downloaded the available RDS file to define brain cell clusters. For defining immune cell clusters we used Seurat and spacexr packages in R [36]. We obtained the appropriate RDS file from Zenodo A Single-Cell Tumor Immune Atlas for Precision Oncology [31].

## Results

**Fig. 1:**
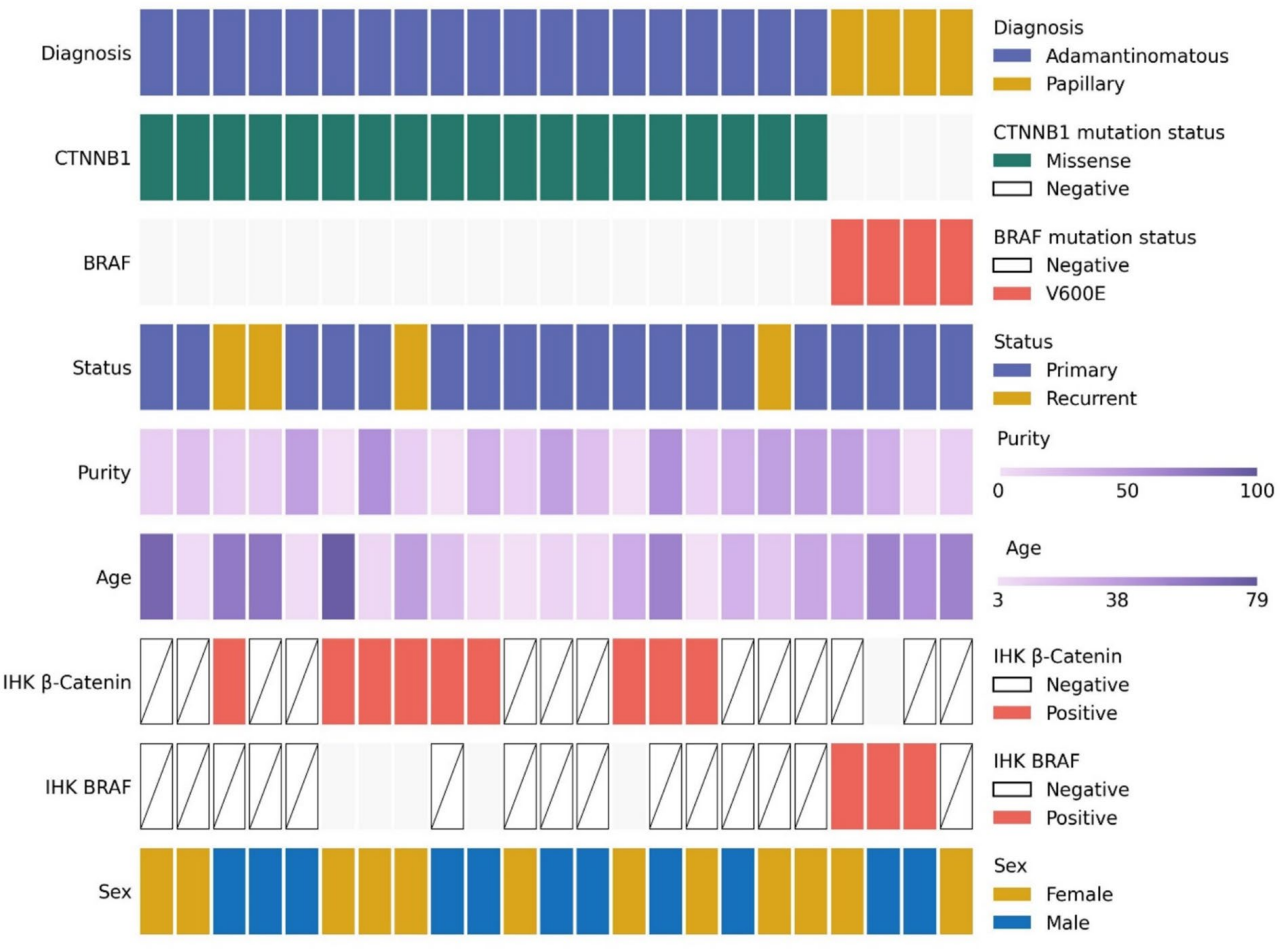
CoMut plot generated using the CoMut package in Python [30] representing demographic, clinical and experimental data for samples in this study

The CoMut plot (Fig. 1) provides a detailed representation of the clinical and molecular characteristics of all 23 CP samples examined in this study. The data reveals a correlation between the diagnosis and the specific mutations identified in the samples, with β-catenin mutations being present in ACP and BRAF p.V600E mutation present in PCP. Among the analyzed samples, four are recurrent tumors, while the remaining 19 are primary tumors. IHC analysis was conducted on nine samples for β-catenin mutations and on two samples for the BRAF p.V600E mutation.

### HE staining and IHC

**Fig. 2:**
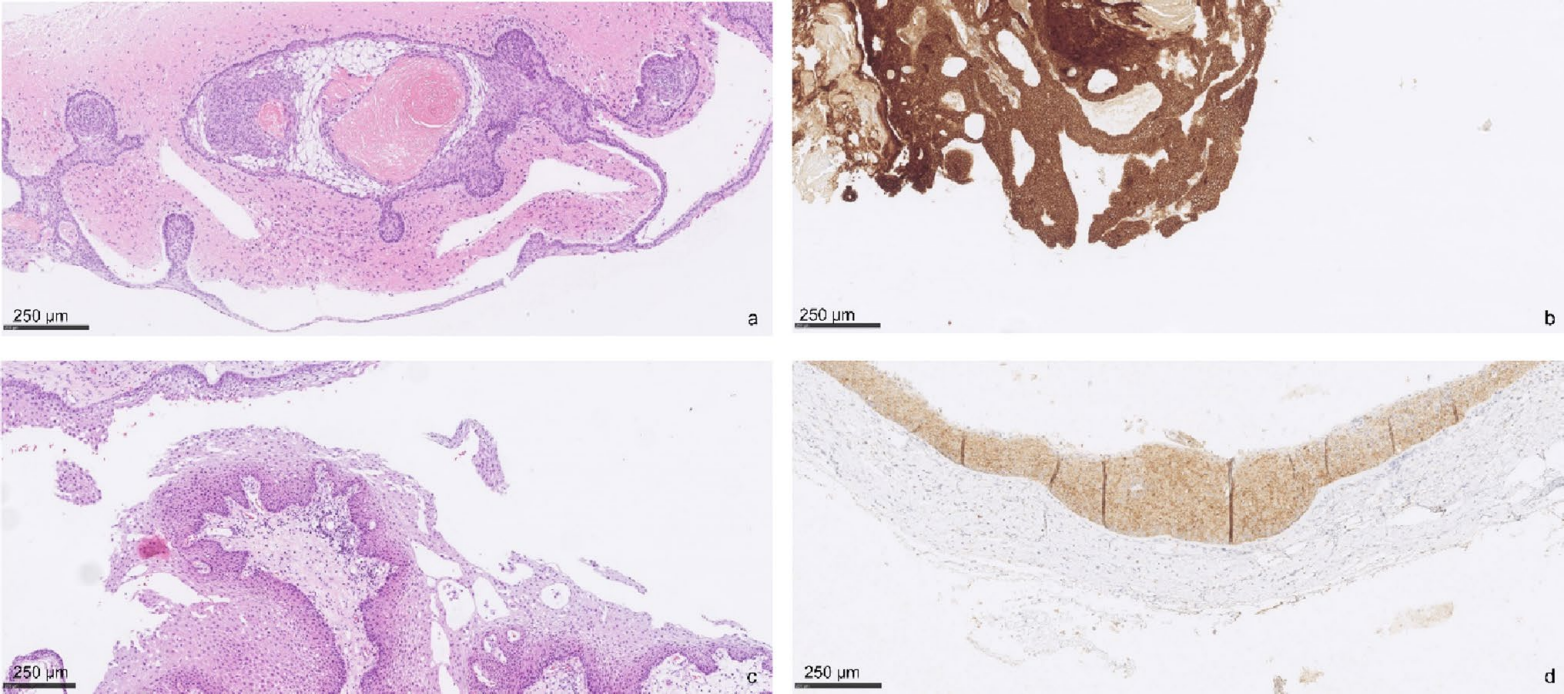
ACP showing typical histologic features, with peripheral palisading, stellate reticulum and ghost cells with brain parenchyma invasion (**a**). β-catenin immunohistochemistry on ACP sample (**b**). PCP showing typical papillary architecture with squamous epithelium and fibrovascular cores (**c**). BRAF p.V600E immunohistochemistry on PCP sample (**d**)

Both groups exhibited typical histologic characteristics and positive IHC for β-catenin or BRAF p.V600E (Fig. 2). Two PCP cases resembled Rathke’s pouch with squamous cell metaplasia, and we resolved this histopathological uncertainty by identifying the BRAF p.V600E mutation through NGS and IHC.

### Somatic mutation detection

Overall, we were able to detect a *BRAF* p.V600E mutation in all four PCP samples; in three with transcriptional analysis and in one, for which data was available, with AmpliSeq technology. All ACP samples carried a somatic *CTNNB1* mutation in exon 3 of the gene, specific mutations can be viewed in Supplementary Table 2. In sample ACP_9, we were unable to detect a mutation using transcriptional analysis, since the tumor content was only 20%. However, we were able to detect the mutation using targeted DNA sequencing. In two ACP samples, the *CTNNB1* mutation was not detected by the CTAT mutation pipeline, although the mutations were visible during manual examination of the *CTNNB1* gene in an Integrative Genomics Viewer (IGV). We confirmed the presence of the mutation in three cases through Sanger sequencing.

### Expression analysis

To understand the differences between PCP, ACP, and healthy pituitary we performed differential gene expression analysis using RNASeqChef [12] with the standard DESeq2 protocol [27]. Examination in SeqMonk indicated that six ACP samples exhibited DNA contamination and were excluded from the differential gene expression analysis. The DEG analysis after elimination included 13 ACP samples, 4 PCP samples and 49 healthy pituitary samples.

We obtained expression data for normal pituitaries as control samples, from the GTEx portal (https://gtexportal.org/home/). We selected pituitary samples as controls because ACP is believed to derive from remnants of Rathke’s pouch, the embryonic precursor of the pituitary.

We detected noticeable differences when comparing healthy pituitary tissues to tumor samples, while we did not observe distinct differences in the expression of genes involved in the the Wnt and MAPK pathways between ACP and PCP. Principal component analysis (PCA) and clustering confirmed the distinction of tumor samples from healthy pituitary.

We specifically focused on the DEG of genes mentioned in the WHO Classification of Tumors of the Central Nervous System (5th ed.) regarding the Wnt signaling pathway, previously studied therapeutic targets, genes involved in healthy pituitary function, genes known to be expressed in ACP and genes coding for keratins.

**Fig. 3:**
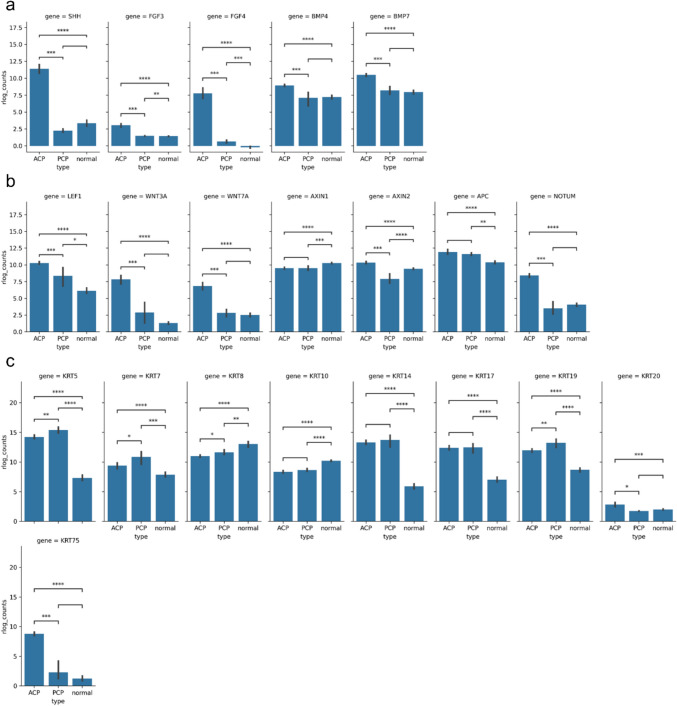
Bar plots representing the statistical evaluation of differential gene expression across selected genes. Genes involved in several signaling pathways (**a**). Genes involved in Wnt/β-catenin signaling (**b**). Keratin genes (**c**). Statistical analysis was performed using a two-sided Mann–Whitney-Wilcoxon test, with significance levels indicated as follows: ns = 0.05 < *p* ≤ 1.00, * = 0.01 < *p* ≤ 0.05, ** = 0.001 < *p* ≤ 0.01, *** = 0.0001 < *p* ≤ 0.001, and **** = *p* ≤ 0.0001 (*ACP* adamantinomatous craniopharyngioma, *PCP* papillary craniopharyngioma)

We observed upregulation of genes encoding several critical signaling factors, including sonic hedgehog (SHH), fibroblast growth factors (FGF), transforming growth factor-beta (TGF-β) and bone morphogenetic proteins (BMPs) (Fig. 3a). Furthermore, several Wnt pathway target genes, including *LEF1, WNT3A, WNT7A, AXIN1, AXIN2, APC*, and *NOTUM*, were upregulated in ACPs (Fig. 3b).

In addition, we observed a significant statistically relevant up-regulation of several keratin genes, including *KRT5, KRT7, KRT8, KRT10, KRT14, KRT17, KRT19* and *KRT20*, in CPs compared to normal pituitary glands (Fig. 3c). These keratins are predominantly soft epithelial keratins. We also observed up-regulation of the *KRT75* gene, a keratin typically associated with hair follicles.

We also observed downregulation of genes involved in pituitary hormone production, including *LHX3, FSHB, GH1*, and *TSHB*. Up-regulation of *TP63* was observed. However, *SOX9* expression varied significantly between ACP and PCP, as well as between PCP and normal pituitary glands.

### Spatial transcriptomic (Xenium)

We performed Xenium-based spatial transcriptomic sequencing on three FFPE sections (two from the ACP group and one from the PCP group). We detected a total of 201,499 cells across all three sections and we determined the expression levels of all 377 genes in the Human Multi-tissue and Cancer Panel.

**Fig. 4:**
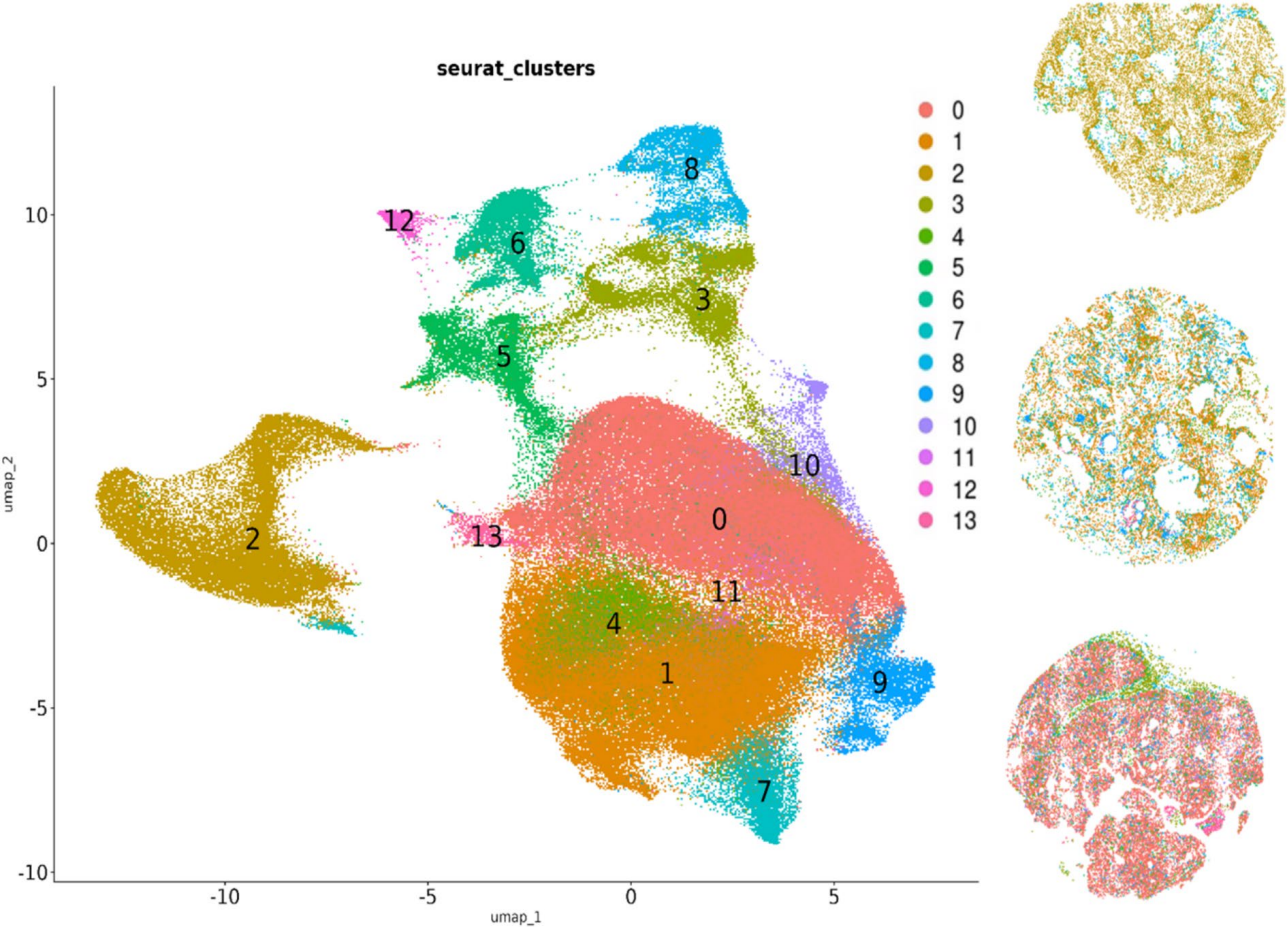
Cellular clustering and spatial localization in craniopharyngioma tissue sections using Xenium spatial transcriptomics. Left: UMAP plot display the distribution of 201,499 profiled cells from two ACP and one PCP. Cells are grouped into 13 distinct clusters based on dimensionality reduction and gene expression profiles from the Human Multi-tissue and Cancer Panel. Right: Adjacent high-resolution spatial maps for each sample depict the localization of the clusters directly on histologic sections of CP samples (top to bottom: one PCP, two ACP). Each cell cluster is color-coded consistently with the UMAP for visual correlation between transcriptional identity and tissue localization

Based on spatial clustering, we reconstructed the presence of 13 cell type clusters (Fig. 4). We performed dimensionality reduction clustering based on the gene expression levels and visualized the distribution in a UMAP plot (Fig. 4).

**Fig. 5:**
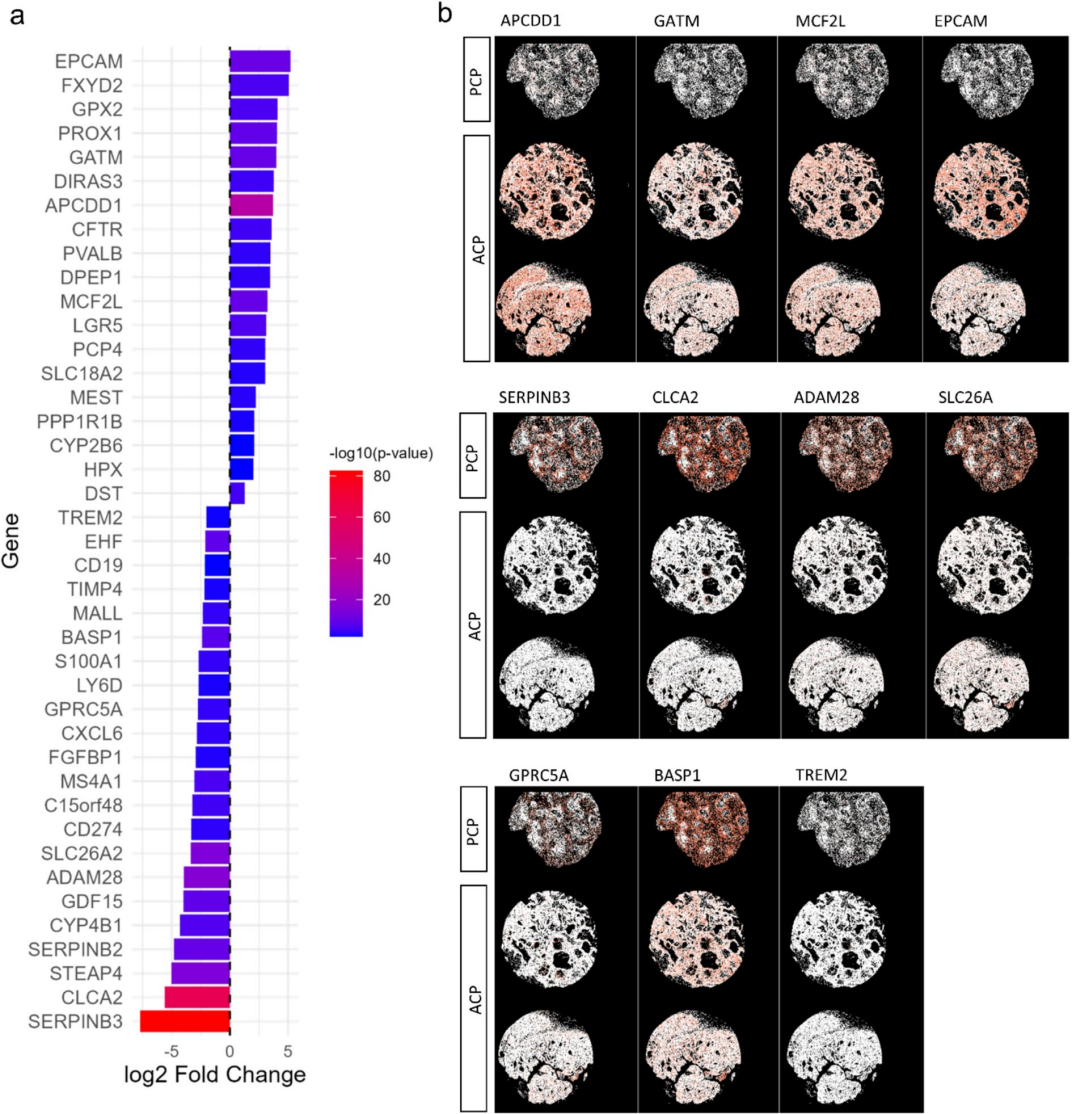
Differentially expressed genes between ACP and PCP obtained from Xenium-based spatial transcriptomics analysis. Bar plot illustrating the log2 fold change of 41 differentially expressed genes between ACP and PCP samples, with upregulated genes shown above and downregulated genes below the axis. Bar colors represent statistical significance, with a color gradient from blue (less significant) to red (highly significant) based on –log10 (*p* value) (**a**). High-resolution spatial distribution maps display selected genes with significant expression differences between ACP and PCP, visualizing localization patterns of four upregulated (APCDD1, GATM, MCF2L, EPCAM) and seven downregulated (SERPINB3, CLCA2, ADAM28, SLC26A, GPRC5A, BASP1, TREM2) transcripts across tissue sections from two ACP and one PCP case. Red intensity indicates greater transcript abundance in spatial context (**b**) (*ACP* adamantinomatous craniopharyngioma, *PCP* papillary craniopharyngioma)

We then obtained information from Xenium Analyzer about transcripts per sample. DESeq2 analysis revealed 41 differentially expressed genes (padj < 0.01) between ACP and PCP (Fig. 5a, b). We observed distinct expression profiles between ACP and PCP, which suggest different biologic behaviors, with implication for tumor growth (upregulated: PPP1R1B, LRG5, MCF2L, PROX1, downregulated: MS4A1, EHF), immune evasion (downregulated: CD274, CXCL6, TREM2, LY6D) and metabolic changes (upregulated: HPX, CYP2B6, DEP1, SLC18A2 and GATM, downregulated: GDF15, CYP4B1 and SLC26A2). Interestingly, we also observed upregulation of the APCDD1 gene (a negative regulator of Wnt signaling) in ACP compared to PCP. High-resolution spatial distribution of selected mRNAs shows clear differences in selected gene expression between the two groups of CPs.

**Fig. 6:**
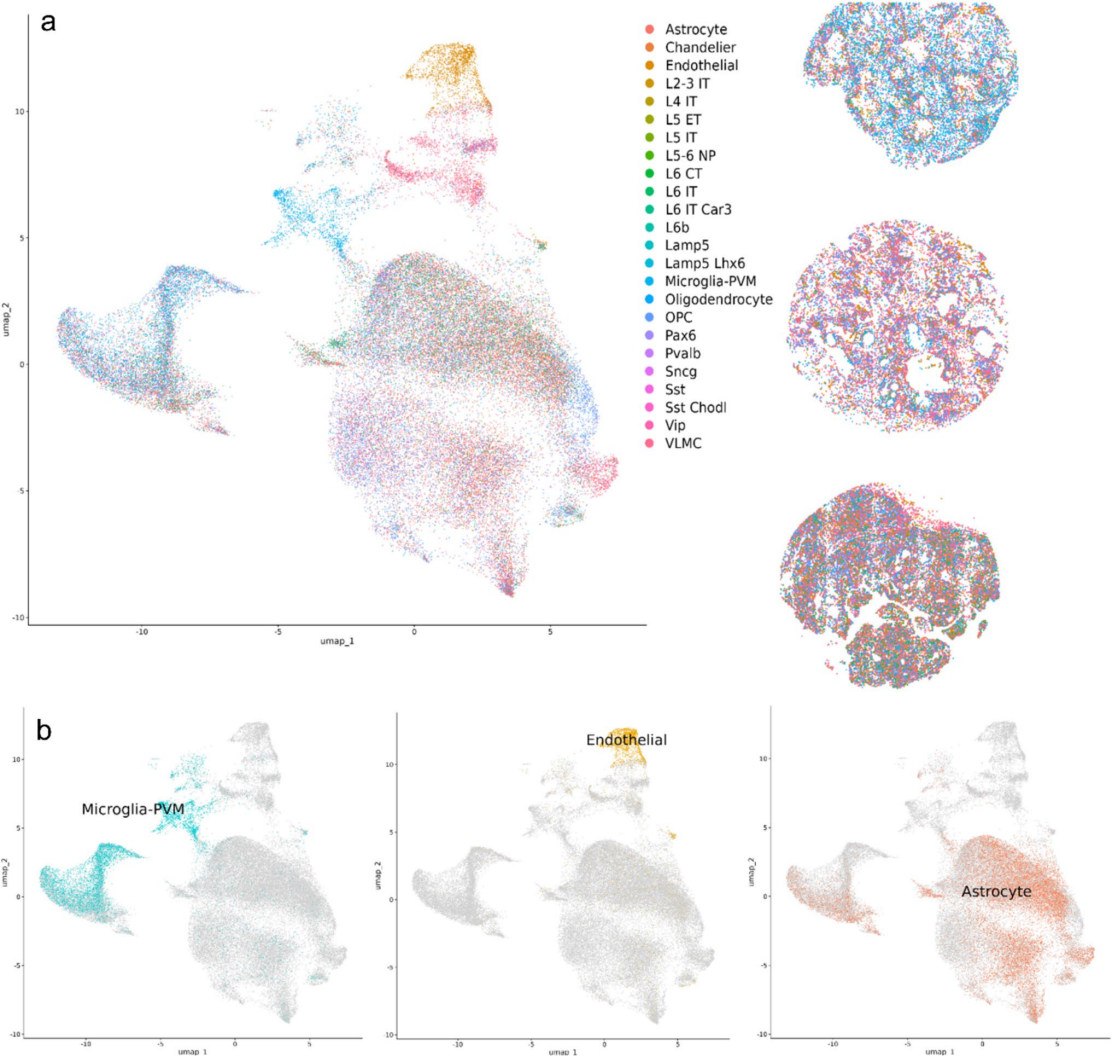
Reference-based clustering and spatial localization of brain cell populations in craniopharyngioma tissues using Xenium spatial transcriptomics. The left panel displays a UMAP plot of reference-based cluster annotation for Xenium-derived transcriptomes, generated by mapping spatial transcriptomic data from ACP and PCP to the Allen Brain Map RNA-Seq Data: Human MTG 10 × SEA-AD reference. Each color represents a distinct cell cluster identified in the tissue, revealing 24 separable clusters including perivascular macrophages (microglia-PVM), endothelial cells, astrocytes, and others. The right panels present high-resolution spatial images of whole tissue slides from PCP and ACP sections, where colored regions reflect the spatial expression and localization of these identified clusters within the tumor and adjacent brain tissue (**a**). Additional UMAP plots highlight the spatial distribution of three selected cell types: perivascular macrophages (microglia-PVM), endothelial cells, and astrocytes (**b**). Cell annotation was performed using existing brain and immune cell atlases due to the limited coverage of the gene panel, and not all clusters could be annotated with complete certainty (*ACP* adamantinomatous craniopharyngioma, *PCP* papillary craniopharyngioma)

We also performed reference-based cell cluster annotation (Fig. 6). We were not able to annotate all the clusters because references for brain tumor, CPs in particular, are not available. Manual annotation was not performed due to the panel only covering 377 genes across different tissue and conditions.

Figure 6 shows the cell clusters obtained using Allen Brain Map RNA-Seq Data: Human MTG 10 × SEA-AD reference. Cell identities in our spatial data were assigned either by direct reference mapping or by correlating marker gene signatures [31]. However, there are limitations to this strategy: external references may not capture tumor-specific cell states or the full diversity present in CPs, and discrepancies in cell type definitions or marker sets across references can affect annotation reliability [31]. In addition, gene panels such as those used in spatial transcriptomics may not cover all canonical markers. Validation with broader gene panels or orthogonal methods will be important for future studies. We were able to identify 24 distinct cell clusters. Perivascular macrophages were shown to be more abundant in PCP than in ACP. Clusters of astrocytes and oligodendrocytes, which are not found in normal pituitary tissue or CPs, can also be observed. These cells could be from the normal brain tissue surrounding the CPs.

We also performed reference-based annotation based on immune cell reference (A Single-Cell Tumor Immune Atlas for Precision Oncology). The most abundant groups of immune cells from the annotation are tumor-associated macrophages (TAMs), as expected in primary brain tumors (Supplementary Fig. 1).

## Discussion

Formalin fixation and paraffin embedding (FFPE) have a negative effect on nucleic acids, leading to RNA fragmentation, cross-linking and reduced RNA yield and integrity. These factors make RNA extraction and downstream applications challenging [8, 30, 37, 44]. Despite these challenges, FFPE samples are a valuable resource for retrospective studies, especially for rare diseases, long-term outcomes or under-researched patient cohorts [8, 30, 37, 44]. In addition, FFPE samples can be stored long-term at low cost, making them ideal for linking RNA expression data to clinical outcomes. While issues such as RNA degradation, fragmentation and low yields can affect the accuracy of transcriptome sequencing, advances in extraction techniques, sequencing technologies and bioinformatics are helping to overcome these limitations [37, 38]. Previous studies [8, 30, 37, 44] have shown that FFPE samples are suitable for RNA isolation and whole-transcriptome sequencing, and our results support these conclusions.

### Expression of genes specified in WHO classification

We observed the overexpression of several keratin genes (*KRT5, KRT7, KRT8, KRT10, KRT14, KRT17, KRT19, KRT20* and *KRT75*) in CPs compared to normal pituitary gland. The keratins overexpressed in CPs are primarily soft epithelial keratins, reflecting the tumor’s origin from the epithelial remnants of Rathke’s pouch. These tumors often include cysts lined by squamous epithelium, which explains the increased keratin expression and supports previous findings [5, 25, 35]. In addition, the adamantinomatous subtype (ACP) commonly exhibits a histopathological feature known as “wet keratin”, which is associated with a high accumulation of keratin proteins. *KRT75*, a hair follicle-specific keratin, is not thought to contribute to the pathology of CPs. However, a previous study [35] demonstrated IHC positivity for human hair keratin in ACP, suggesting possible follicular differentiation. Generally, the expression of keratin genes is low in normal pituitary gland, except KRT8, KRT10 and KRT18, according to a study researching tissue-specific expression patterns of keratin genes [18]. Understanding keratin expression profiles may enhance the diagnosis of CPs, especially when limited tissue samples are available. Recent single-cell and spatial studies confirm that ACPs exhibit cellular diversity, encompassing classic epithelial and whorl-like cells as well as senescent and stem-like tumor cell populations. Our findings of distinct keratin gene expression and Wnt pathway activity are supported in a recent study [39], which highlighted the spatial arrangement and potential functional roles of these diverse cell types.

In addition to the keratin overexpression, we observed elevated expression of genes encoding several important signaling factors, including sonic hedgehog (SHH), fibroblast growth factors (FGF), transforming growth factor-beta (TGF-β) and bone morphogenetic proteins (BMPs). These factors are secreted by epithelial whorls, which are a hallmark of ACP [2, 3, 9, 43]. Notably, ACPs exhibited evidence of active hedgehog signaling, as shown by the significant overexpression of SHH compared to normal pituitary gland and PCP. The overactivation of SHH signaling may be linked to cross-regulatory interactions between the hedgehog and Wnt/β-catenin pathways, which are known to influence each other. Moreover, our results show upregulation of several targets of the Wnt signaling pathway, including *LEF1, WNT3A, WNT7A, AXIN1, AXIN2, APC,* and *NOTUM*, further supporting the involvement of Wnt/β-catenin signaling in ACPs. The upregulation of LEF1, a transcription factor activated by β-catenin, highlights the pathway’s contribution to the transcriptional changes seen in ACPs. Similarly, overexpression of WNT3A and WNT7A, key ligands in the Wnt signaling pathway, likely enhances the activation of downstream Wnt signaling cascades. ACP, an important tumor suppressor gene, and NOTUM, a Wnt inhibitory factor, were similarly upregulated, further reflecting dysregulation of Wnt signaling in ACPs.

We also observed downregulation of genes related to pituitary hormone production, including *LHX3, FSHB, GH1,* and *TSHB*, all of which play an important role in regulating pituitary function. *LHX3* is a critical transcription factor required for pituitary development and the expression of various pituitary hormones. *FSHB* encodes for the beta subunit of FSH, which plays a vital role in reproduction. *GH1* is a key gene that encodes the growth hormone, which is crucial for growth and metabolism, while *TSHB* encodes the beta subunit of TSH, which is essential for the regulation of thyroid function.

The WHO CNS Tumor Classification also specifies the expression of p63 in epithelial layers when determining the immunophenotype. Our DGE supports this, since the *TP63* gene was upregulated in ACP and PCP compared to normal pituitary gland. The WHO CNS Classification also states variable expression of SOX2 and SOX9. In our study, we observed only a weak statistical difference in expression of SOX2 and SOX9 between normal pituitary tissue and ACP. However, SOX9 expression varied significantly between ACP and PCP and between PCP and normal pituitary gland. These results are consistent with the complex immunophenotypic profile of CPs and further support the diagnostic utility of these markers.

The detection of *BRAF* p.V600E mutations in all PCP samples is consistent with previous studies and underlines the role of the MAPK signaling pathway in the pathogenesis of PCP. This mutation is a potential target for molecular therapies with BRAF inhibitors, which are already the standard of care in other cancers. All ACP samples had mutations in exon 3 of the *CTNNB1* gene, which is consistent with activation of the Wnt signaling pathway. The presence of these mutations in all ACP samples confirms their important role in tumor development and progression. Interestingly, our study found that some mutations were only detectable by specific sequencing technologies, such as RNA sequencing and targeted DNA sequencing, emphasizing the importance of using comprehensive molecular techniques for accurate mutation detection, and critical evaluation of methods used for mutation detection. One of the most notable observations was the occurrence of specific CTNNB1 mutations in recurrent ACP samples, suggesting that these mutations could be a prognostic factor in defining tumor recurrence, as suggested in previous studies [13, 15]. This could lead to improved monitoring and management of patients harboring these mutations, and potentially assist in the early detection of ACP recurrence. However, further investigations in a larger patient cohort are needed to confirm the prognostic significance of these specific mutations. We also recommend conducting longitudinal studies to examine how such mutations evolve over time and contribute to tumor progression and recurrence.

Interestingly, among the six ACP samples that either presented as recurrent tumors or where the patients developed ACP again later in life, five exhibited two distinct mutations in the CTNNB1 gene: T41I and S37Y. Notably, mutations affecting T41 have previously been linked to a poorer prognosis [13]. This association suggests that specific alterations in the CTNNB1 gene, particularly those involving T41, may contribute not only to the recurrence of ACP but also to the overall severity and progression of the disease.

### Expression of genes based on the Xenium human multi-tissue and cancer gene panel

To our knowledge, this is the first study to utilize spatial transcriptomic based analysis for CPs. Spatial transcriptomics was chosen to capture the spatial heterogeneity of gene expression within CPs, providing insights that bulk transcriptomics could not reveal, and enhancing our understanding of tumor biology, pathology and therapeutic potential [4, 23, 26]. Our Xenium-based spatial transcriptomics analysis was limited to 377 genes included in the Human Multi-tissue and Cancer Panel. Even with this limitation, we were able to show different expression profiles between ACP and PCP. Clustering based on gene expression levels revealed 13 distinct cell groups, some of which we were able successfully to annotate using a publicly available reference.

Xenium analysis revealed 41 differentially expressed genes between the ACP and PCP groups. Notably, genes associated with tumor growth, such as PPP1R1B and PROX1, were upregulated in the ACP group, indicating a possible enhancement of tumor proliferation and survival mechanisms. Conversely, downregulation of MS4A1 and EHF may suggest an immune escape strategy employed by ACP tumors. Furthermore, the immune evasion observed in ACP, marked by downregulated genes such as CD274, aligns with recent findings in a study [45], which reported fewer T cells and an increased infiltration of immune-suppressing macrophages in ACP. This immune landscape further supports the potential for targeted immunotherapies in ACP. In addition, another study [7] demonstrated that AXL signaling contributes to immune escape. AXL is a receptor tyrosine kinase that promotes immune suppression, cell survival, and tumor progression. Inhibition of AXL was shown to enhance immune responsiveness, highlighting its potential as a therapeutic target. These findings highlight how both immune cells and tumor signals help ACP evade the immune system, pointing to new treatment possibilities. Furthermore, the metabolic alterations observed in ACP, highlighted by the upregulation of HPX and CYP2B6, point to the adoption of unique metabolic pathways by the tumor. The small sample size for PCP (*n* = 4 transcriptomic analysis, *n* = 1 spatial transcriptomics) reduces statistical power, especially for immune and microenvironment analyses. As shown in studies [4, 23], robust characterization of immune cell diversity and spatial architecture in rare tumors requires larger cohorts for reliable conclusions. Therefore, our conclusions regarding immune signatures, tumor biologic behavior, and microenvironment features in ACP and PCP should be viewed as preliminary and hypothesis-generating, pending further validation in larger, more comprehensive studies.

Interestingly, APCDD1 was upregulated in ACP when compared to PCP. APCDD1 is a membrane protein that acts as a negative regulator of Wnt signaling by lowering the concentration of available Wnt ligands [20]. APCDD1 is known to be abnormally expressed in colon cancer and Wilms’ tumors [46]. However, the role of APCDD1 in the biology of CPs has not been yet described. Despite its inhibitory function, APCDD1 is a target of Wnt signaling and thus gets upregulated when Wnt is hyperactive. The upregulation represents an attempt to balance the excessive Wnt activity caused by CTNNB1 mutations. Recent study [20] shows that APCDD1 limits Wnt signaling by directly binding and neutralizing lipid-modified Wnt ligands at the cell surface. Its upregulation in ACP likely reflects a negative feedback response to persistent Wnt pathway activation, but this mechanism may be insufficient to fully counteract oncogenic signaling. This complex interplay highlights the regulatory challenges in Wnt-driven tumors such as ACP, although it is also possible that these differences are not only because of the Wnt/β-catenin pathway, but are a consequence of changed regulation of different branches of Wnt signaling.

### Limitation and future perspectives

Our application of spatial transcriptomics to CPs provides a valuable layer of insight that complements high-resolution single-cell data and functional studies like [7, 39, 45]. Integrating spatial and molecular profiling with clinical outcomes, as presented in these recent works, will be critical for translating biomarker findings into improved diagnostics and personalized therapies, particularly those targeting pathways such as Wnt, AXL, and the tumor immune microenvironment. Future studies should link spatial cellular context and molecular profiles with treatment response and prognosis in larger well-annotated cohorts.

We would also like to mention obstacles we encountered in this study so that other researchers can take them into account when planning their research. FFPE samples are an important, and often the only source of RNA, especially in rare tumors, and must be considered for transcriptional analysis. The successful extraction and sequencing of RNA from FFPE samples has shown that it is possible to use archival tissue for molecular studies, which is crucial for the investigation of rare diseases with limited sample availability. DNA contamination was detected in six samples, even though DNase treatment was performed during the isolation step. For this reason, we recommend that, if possible, all samples for cDNA library preparation are additionally treated with DNase prior to any other steps.

Further validation by IHC studies and additional functional analyses of the mentioned genes could provide deeper insights into their role in CPs. Exploration of therapeutic interventions targeting the Wnt, SHH or other signaling pathways may offer new treatment options, especially in cases in which surgical resection is challenging. Despite the significant findings, our study is limited by the small sample size, due to the rarity of CPs. Larger studies are needed to validate these results and further explore the molecular mechanisms underlying these tumors.

The consistent presence of BRAF p.V600E mutations in PCP and *CTNNB1* exon 3 mutations in ACP emphasizes the unique pathogenesis of each subtype. The identification of specific mutations and distinct gene expression profiles provides a foundation for developing targeted therapies and improving diagnostic accuracy. Despite the challenges of working with FFPE samples and the limited sample size, our research lays the groundwork for future studies to improve diagnosis and treatment strategies for these rare but impactful tumors. In addition, our study is one of the first to look at spatial transcriptomic of these tumors, providing deeper insight into the molecular background. This study underscores the importance of comprehensive molecular profiling in rare tumors and highlights the potential for personalized medicine in the management of CPs.

## Supplementary Information

Below is the link to the electronic supplementary material.

Supplementary file1 (PDF 952 KB)

## Data Availability

The raw data supporting the conclusions of this article will be made available by the authors on request.
