# Supplementary material for: Comprehensive molecular characterization of craniopharyngiomas using whole transcriptome and spatial transcriptomics approaches
Source: Brain Tumor Pathol. 2025 Jul 9;42(4):130–42. doi: 10.1007/s10014-025-00509-z (PMC12518426; doi:10.1007/s10014-025-00509-z)
Supplement: Supplementary file 1 — Supplementary file1 (PDF 952 KB) [file 10014_2025_509_MOESM1_ESM.pdf]

Supplementary Material:

Supplementary Table 1 *Primers sequences used in Sanger sequencing.*

Primer	Sequence
CTNNB1_e3.1_F_DNA	CCAATCTACTAATGCTAATACTGTTTCG
CTNNB1_e3.1_R_DNA	TCTTCCTCAGGATTGCCTTTAC
CTNNB1_e3.2_F_DNA	ACTACCACAGCTCCTTCTCT
CTNNB1_e3.2_R_DNA	ATTCTGACTTTCAGTAAGGCAATG
CTNNB1_e3.1_F_RNA	TGGCTACTCAAGCTGATTTGAT
CTNNB1_e3.2_R_RNA	TGAGCTCGAGTCATTGCATAC

Supplementary Table 2 *Summary of detected mutations in ACP samples and methods used for mutation detection. *mutation observed only in IGV.*

Sample	Mutation in *CTNNB1* gene	Method of detection
ACP_1	c.104T>G, p.Ile35Ser	RNA-Seq
ACP_2	c.110C>T, p.Ser37Phe	RNA-Seq
ACP_3	c.122C>T, p.Thr41Ile	RNAseq* + Sanger sequencing
ACP_4	c.110C>A, p.Ser37Tyr	RNA-Seq
ACP_5	c.100G>A, p.Gly34Arg	RNA-Seq
ACP_6	c.122C>T, p.Thr41Ile	RNA-Seq
ACP_7	c.97T>C, p.Ser33Pro	RNA-Seq
ACP_8	c.122C>T, p.Thr41Ile	RNA-Seq
ACP_9	c.110C>T, p.Ser37Phe	targeted DNA sequencing
ACP_10	c.94G>T, p.Asp32Tyr	RNA-Seq
ACP_11	c.100G>A, p.Gly34Arg	RNA-Seq
ACP_13	c.98C>T, p.Ser33Phe	RNA-Seq
ACP_14	c.101G>A, p.Gly34Glu	RNA-Seq
ACP_15	c.122C>T, p.Thr41Ile	RNA-Seq
ACP_16	c.110C>T, p.Ser37Phe	Sanger sequencing
ACP_17	c.98C>T, p.Ser33Phe	RNAseq* + Sanger sequencing
ACP_18	c.101G>A, p.Gly34Glu	RNA-Seq
ACP_19	c.110C>T, p.Ser37Phe	RNA-Seq
ACP_20	c.121A>G, p.Thr41Ala	RNA-Seq

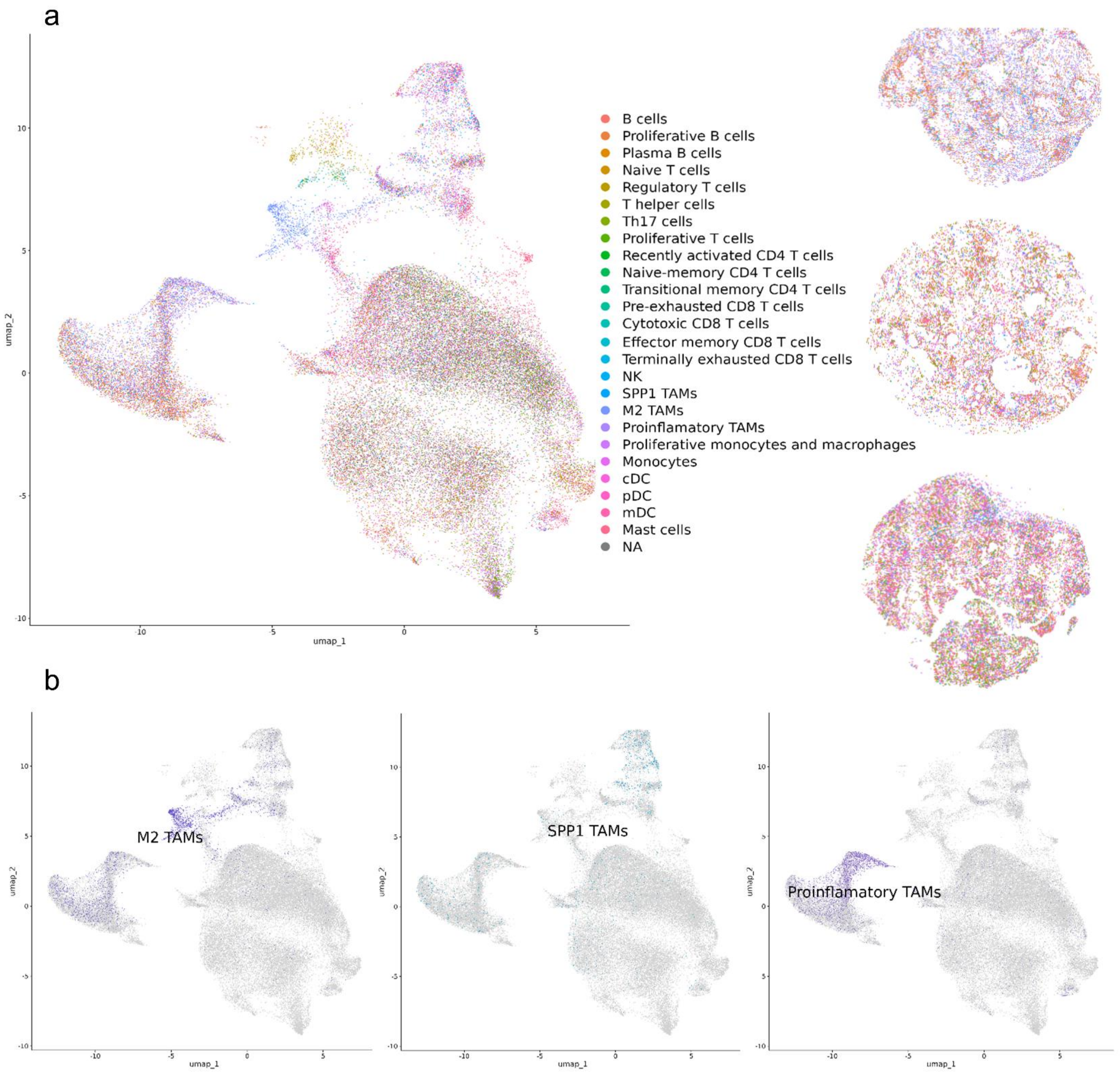

Supplementary Figure 1: *Xenium-Based Spatial Transcriptomics Analysis of ACP and PCP samples. Left is UMAP with distinct immune cell groups based on a reference annotation approach (reference: A Single-Cell Tumor Immune Atlas for Precision Oncology). Right are high-resolution image of whole sample slides (top to bottom: PCP, ACP and ACP) representing the same clusters. Additionally, we show three UMAP plots representing three specific cell clusters obtained with the reference annotation.*
